# The Effects of Rudeness on NICU Medical Teams Studied by a New Tool for the Assessment of Decision-Making Group Dynamics

**DOI:** 10.3390/children9101436

**Published:** 2022-09-21

**Authors:** Yarden Riskin, Arieh Riskin, Hussein Zaitoon, Clair Habib, Einav Blanche, Ayala Gover, Alex Mintz

**Affiliations:** 1 Interdisciplinary Center (IDC), Reichman University, Herzliya 4610101, Israel; 2 The Faculty of Industrial Engineering & Management, Haifa 3200003, Israel; 3 Technion, Israel Institute of Technology, Haifa 3200003, Israel; 4 Departments of Neonatology and Pediatrics, Bnai Zion Medical Center, 47 Golomb Street, P.O.B. 4940, Haifa 31048, Israel; 5 Ruth & Bruce Rappaport Faculty of Medicine, Haifa 31096, Israel; 6 Genetic Institute and Pediatric Metabolic Unit, Rambam Healthcare Campus, Haifa 3109601, Israel

**Keywords:** Groupthink, Polythink, decision-making group dynamics, medical teams, rudeness

## Abstract

Background: Group decision-making can be placed on a continuum of group dynamics, between Groupthink and Polythink. Objective: To present a new assessment tool for the characterization of medical teams’ decision-making group dynamics, and test it to study the effects of exposure to rudeness on various types of group dynamics. Methods: Three judges who watched videotapes of critical care simulations evaluated 24 neonatal intensive care unit teams’ decision-making processes. Teams were rated using the new assessment tool, especially designed for this quantitative study, based on items adapted from symptoms of Polythink and Groupthink. Results: Measures of reliability, inter-rater agreement and internal consistency, were reasonably good. Confirmatory factor analysis refined the tool and verified that the symptoms in each category (Polythink or Groupthink) of the refined 14 items’ assessment tool were indeed measures of the construct. The average General Score was in the range of the balanced dynamic on the continuum, and without tendency towards one of the extremities (Groupthink or Polythink). No significant effect of exposure to rudeness on group dynamics was found. Conclusions: This is a first attempt at using quantitative methods to evaluate decision-making group dynamics in medicine, by adapting symptoms of Groupthink and Polythink as items in a structured assessment tool. It suggests a new approach to understanding decision-making processes of medical teams. The assessment tool seems to be a promising, feasible and reasonably reliable research tool to be further studied in medicine and other disciplines engaged in decision-making.

## 1. Introduction

In recent years, the awareness to rudeness in workplaces has grown, and there are many studies about the effects of exposure to this kind of behavior on individuals and groups [1,2,3,4,5]. Rudeness is defined as an insensitive or impolite behavior, which is enacted by a person who shows disrespect or inconsideration towards others. Few studies have found that direct and indirect experiences of rudeness from different sources hamper individuals’ task execution and performance. In addition, rudeness led to deterioration in helpfulness to others, and negatively affected flexibility, creativity and cognitive performance. Another finding regarding effects of rudeness is a spill-over effect on bystanders who were not involved in the original interaction [1]. Other studies found that rudeness in the workplace, embodied by different sources, led to deteriorated performance, negatively affected creativity, social behavior and cognitive processes, as well as increased aggressive thoughts in onlookers to these behaviors. Therefore, rudeness in the workplace can have harmful and serious effects—performance, behaviors and atmosphere can be negatively affected [2]. 

Rudeness is a very common phenomenon in the medical world, embodied by patients, their families, colleagues, external experts, etc. Studies have found it can have harmful effects on diagnosis and treatment [4,5]. Furthermore, rudeness exhibited by an external authority figure might negatively affect information sharing in a medical team, and consequently effect diagnostic performance, meaning the diagnostic process. Moreover, rudeness can negatively affect help sharing and giving assistance in the team, leading to deterioration in procedural and technical performances, which are part of the treatment [4]. The performance of teams, which were exposed to rudeness, was poorer, and the sharing processes which usually allow medical teams to overcome individual performance deficits were damaged as well [5].

Researchers also found that rudeness represents a significant social threat [3], which can lead to different reactions from the team facing it—it can lead to greater internal cohesion and conformity [6], or it can lead to the opposite result of less cohesion, more disagreements and difficulty reaching a consensus [7].

The nature and work environment in the medical world has unique characteristics, such as teamwork, stress situations, and multiple decision-making on a daily basis. Due to the high rate of medical errors, and the severe consequences that may result from them for patients and their families, it is important to examine the group decision-making dynamics of medical teams. Better understanding of medical teams’ group dynamics may help improve the quality of healthcare services, and the culture of discourse in the medical world.

**Figure 1:**
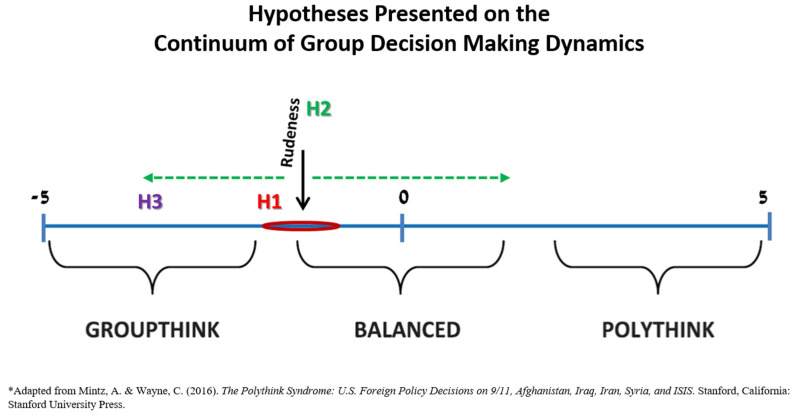
Hypotheses presented on the continuum of group decision-making dynamics. Adapted from Mintz A & Wayne C (2016) [8]. The Polythink Syndrome: US Foreign Policy Decisions on 9/11, Afghanistan, Iraq, Iran, Syria and ISIS. Stanford, California: Stanford University Press with permission.

Group decision-making can be placed on a continuum between Groupthink and Polythink. Groupthink is a theory which claims there are certain situations where a group leans towards conformity in its decision-making [8,9,10], while Polythink is a theory which postulates that there are situations where a multitude of opinions and views in a group exist, resulting even in intra-group conflict and dissent [11,12]. These two theories represent the extreme ends on the group dynamics continuum, while Convergence-Divergence (Con-Div) is placed at the center and is the balanced and often the optimal situation [11,12] (Figure 1). Polythink is a theory, which deals with situations where a multitude of opinions and views in a group may even result in intra-group conflict and dissent. These conditions make it difficult to reach a consensus regarding the problem they are facing and to the best solution for it. Therefore, in extreme cases Polythink may lead to suboptimal decisions, and even decision paralysis [11,12]. In contrast, Groupthink is a theory, which claims there are certain situations where a group leans towards conformity in its decision-making. These situations happen when the group members work closely with one another, share the same values and are faced with an extreme situation or crisis, which puts them under tremendous pressure. In these conditions, the feeling of group pressure leads the members to be less critical and to abstain from casting doubts or arguing, as well as to greater motivation to reach a consensus on every issue. The closer and more collegial a group, the greater the danger for Groupthink, and in extreme cases it may lead to bad decisions [9,10]. Groupthink is a phenomenon which is relevant and exists among medical teams [8]. Teamwork is very common in medicine, and these teams make many decisions every day. Moreover, these teams are usually small and their members know each other and develop close working relations. Therefore, there is often internal cohesion in these teams, and the danger of a tendency for Groupthink in their decision-making is high [8,13]. This tendency may be further enhanced due to the nature of work in some of the medical units. In professions where the work includes critical care and life-threatening conditions, like in the NICU, the teams face stress situations, which are due to both the clinical condition they are treating and the time pressure. One example is the emergency situation of resuscitation. According to Janis [9,10], stress and extreme situations may increase a group’s tendency for conformity and Groupthink. Decision-making Con-Div refers to a decision-making dynamic in which the group members offer diverse opinions and options for deliberation, yet at the same time agree on the same greater goals and vision [11]. In the medical world there is a tendency towards Groupthink, due to the close work of small teams made up of members who know each other [8,13], but the existence of Polythink among medical teams has not yet been studied.

In this study, we examine the effects of rudeness (exhibited by an external authority figure) on the group dynamics of neonatal intensive care unit (NICU) teams’ decision-making process during the critical care of a preterm infant [4]. Specifically, in this study we try to evaluate where medical teams are placed on the group dynamics continuum between Groupthink and Polythink, and whether exposure to rudeness affects their position and moves the dynamics towards one of the two ends (Figure 1). For a group experiencing this kind of behavior, rudeness represents a significant social threat [3]. Therefore, there is a possibility it might affect inter-group dynamics. Studies show that people react differently to threats, and similarly groups react in different ways to threats. Most researchers believe that when a team which already has some degree of internal cohesion deals with an external threat, the members have a greater tendency to consolidate and unite together against it [6]. Few researchers disagree, and claim that although on one hand, an external threat might lead to greater cohesion in the team, pressure to conform and a desire to reach consensus; on the other hand, it can actually lead to the opposite result, of less cohesion, more disagreements and instability, as well as difficulties reaching consensus in the team [7]. The reaction of teams to social threats (such as rudeness) is not clear, and there is disagreement whether it would lead to greater unity and conformity or to less cohesion and more dissentions among group members [3,7]. The findings about the effects of rudeness on medical teams do not assist in clarifying the picture. The decrease in information sharing can be an indication of a tendency towards Groupthink, if it is an expression of the self-appointed “mindguards” symptom or self-censorship of team members; but it could also indicate that there is a tendency towards Polythink, if it expresses a lack of communication or a certain framing effect. Similarly, the negative influence on seeking and offering of assistance in a team can be an indication of a tendency towards Polythink, only if it is an expression of differing opinions and conflicts in the team or decision paralysis. Therefore, there is no consensus among researchers in regard to how an external threat (such as rudeness) will affect a team in general, and specifically a medical team. It could lead to greater unity and conformity in the team, and a tendency towards Groupthink or not.

Thus, we postulated three hypotheses that we examined in this study (Figure 1):

**Hypothesis** **(H1).** *NICU teams not exposed to rudeness will fall around the range of Con-Div, with a tendency towards Groupthink.*

**Hypothesis** **(H2).** *Exposure to rudeness will lead to more prominent expressions of either Groupthink or Polythink symptoms in the teams.*

**Hypothesis** **(H3).** *Exposure to rudeness will have a more prominent effect on enhancing Groupthink symptoms than Polythink.*

We have developed a new quantitative assessment tool for the characterization of medical teams’ decision-making group dynamics. Teams were rated using this assessment tool, based on items adapted from the symptoms of Polythink and Groupthink. To test the validity and feasibility of this new assessment tool we used data gathered in a previous study regarding the effects of exposure to rudeness on medical teams [4]. Previous studies used qualitative research methods to test group dynamics’ theories. Moreover, this is the first study to identify intra-group dynamics in medical teams beyond Groupthink, and apply it to Polythink and Con-Div. This study presents the first attempt at using a quantitative research assessment tool to test the theories in the field of decision-making dealing with the different types of group dynamics, by adapting the symptoms described in the literature into a structured assessment tool.

## 2. Methods

The study was a quantitative research, based on a structured assessment tool that the judges were asked to fill about each participating team’s group dynamic. The assessment tool was built based on the symptoms of Polythink and Groupthink [8,9,10,11,12], and the symptoms were adapted, with the help of a senior neonatologist, for the evaluation of medical teams. Each symptom appeared as an individual item for the judges to rate, using a 5-point Likert scale (from 1 = strongly disagree to 5 = strongly agree) (Table 1).

Scoring 1 on any given item indicates proximity to the range of Con-Div. As the score increases, so does the movement on the group dynamics continuum further away from Con-Div and closer to one of the two ends—Polythink or Groupthink—according to the items evaluated (Figure 1).

The research was based on a reexamination and reevaluation of data collected in an earlier study [4]. Specifically, videotapes of 24 NICU teams from different hospitals in Israel that participated in simulation sessions during 2013–2014 were reviewed. The teams were faced with a life-threatening condition of a preterm infant with necrotizing enterocolitis. Each team included one physician and two nurses from the same unit [4]. During the simulation, the teams received comments from an external expert, which were scripted and pre-recorded yet seemed live to the participants. The teams were randomly assigned to either exposure to rudeness from the expert, or control. Accordingly, they received comments containing mildly rude statements unrelated to their performance, or neutral comments [4]. 

In this study, three independent judges, all of whom are pediatricians who regularly work in the NICU, reexamined the videotapes and filled the assessment tool regarding each team. Before reviewing the videotapes, each one of the three judges had a training encounter with the second author to go over the assessment tool and clarify each of the definitions with examples for grading. Then, each judge watched the recorded simulations and assessed the teams individually and independently, according to the structured assessment tool. The videotapes were shown to each judge in the same randomized order, and all of them were edited to protect the participants’ identities. In addition, the judges were given materials relevant to the simulation and to the current study. The IDC’s Institutional Review Board approved the study on 23 September 2019 (No approval code provided).

Statistical analyses were conducted using JMP pro version 14.0.0 (SAS Institute Inc., Cary, NC, USA), SigmaPlot, version 11.0 (Systat Software Inc., San Jose, CA, USA), R version 2.15.0 (The R Foundation for Statistical Computing), and MPlus, version 7.2 (Muthen & Muthen, Los Angeles, CA, USA).

As each team’s group dynamics was rated by three judges, reliability was measured for internal consistency using Cronbach’s α and for inter-rater agreement by intraclass correlation coefficient (ICC(1)). Based on theoretical grounds we grouped the 15 items of the original assessment tool into two factors that were consistent with Groupthink and Polythink symptoms. Then, we employed confirmatory factor analysis (CFA) using a 2-factor model to test whether the items in each category were indeed measures of the construct. Comparisons of Polythink, Groupthink and general scores in the rudeness and control groups were conducted by using Student’s t test. 

## 3. Results

Altogether, we had 72 evaluations by three judges for each of the various symptoms presented in the assessment tool. Inter-rater agreement was good for most of the items in the assessment tool—ICC(1) in the range of 0.5–0.84, and for each set of items comprising the Polythink and Groupthink symptoms (0.66 and 0.82, respectively) (Table 2).

Fourteen out of the 15 items in the original assessment tool were grouped into two factors that were consistent with Groupthink and Polythink symptoms (item 14 was removed because it was not related to any of the factors, and did not represent a Polythink symptom as it was supposed to). Then, we employed confirmatory factor analysis (CFA) using a 2-factor model to test whether the items in each category were indeed measures of the construct. Overall the measures for the whole model fit of the CFA were good when seven items were related to each of the factors of Groupthink and Polythink symptoms. Most of the standardized estimates of the factor loadings for the different items in each group were high (0.7 or more) with high percentage of explained variance (R2 36–82% for Polythink and 21–45% for Groupthink) and statistically significant *p* values (<0.001) (Table 2). Only items 4 and 7 loadings on Polythink and items 8 and 15 loadings on Groupthink had lower estimates explaining less of the variance in each factor, although still statistically significant. Thus, we have tried to test CFA on a model of a shorter 10 items’ assessment tool, consisting of only 5 items in each of the two factors, but the model fit for this shorter assessment tool was less optimal.

Cronbach’s α was calculated for the 7 items included in each group dynamics (Table 2), respectively: Polythink Cronbach’s α = 0.79 and Groupthink Cronbach’s α = 0.68. Thus, there was sufficient reliability to aggregate the items in each of the two sets—Polythink and Groupthink. 

The sets were created by averaging the scores of each relevant item evaluated in each team, across all three judges. Each team received a separate score for Polythink (average for all teams 2.86 ± 0.80) and a score for Groupthink (average for all 2.74 ± 0.65), according to the average calculated from all three judges. As mentioned before, group decision-making dynamics can be placed on a continuum between Groupthink and Polythink, with the balanced range of Con-Div in the center [11,12]. Based on this logic, each team received a General Score calculated by subtracting the Groupthink score from the Polythink Score (average for all 0.12 ± 0.65). T tests were conducted to compare the means of the Polythink and Groupthink scores, separately, between the rudeness and control groups. No difference was found between the teams who were exposed to rudeness and those who were not, in either Polythink or Groupthink sets. In addition, no difference was found between the mean general scores of the rudeness and control groups (Table 3).

## 4. Discussion

Although this study did not find the hypothesized effects of exposure to rudeness on medical teams’ group dynamics in decision-making, it showed that the suggested quantitative method of research could be useful. The assessment tool allowed the judges to evaluate group dynamics of the participating teams from the recorded simulations they watched with acceptable reliability (both relative consistency among judges and internal consistency within each set of group items). CFA verified that the items (regarding symptoms) in each category of the improved 14 items’ assessment tool were indeed measures of the construct (Polythink or Groupthink).

In this study we examined how rudeness, exhibited by an external authority figure (in this case a foreign expert), affected NICU teams’ decision-making process, i.e., their group dynamics. We did so by examining our new assessment quantitative tool of group dynamics on NICU teams’ decision-making process during simulations of stressful critical care of a sick preterm infant [4].

Based on the discussion above, we suggested three hypotheses. The first hypothesis (H1) addressed the group dynamics continuum, and specifically Convergence-Divergence (Con-Div) placed at its center [11,12]. We suggested that the NICU teams not exposed to rudeness will fall around the range of Con-Div, with some tendency towards Groupthink, because of the characteristics of medical teams acting in emergency life-threatening situations [9,10]. The average General Score calculated for the 24 teams examined in this study was in the range of Con-Div, i.e., around the balanced dynamic and without tendency towards one of the extremities. NICU teams not exposed to rudeness (control group) did fall around the range of Con-Div, close to the score 0. However, a tendency towards Groupthink was not detected. Therefore, this hypothesis was only partially supported. The second hypothesis (H2) was based on the assumption that exposure to rudeness represents a social threat to the team [3]. This could lead to either more unity [6] and a tendency towards Groupthink, or work in the opposite direction leading to less cohesion and more disagreement [7] that might cause Polythink. Thus, we suggested that exposure to rudeness will lead to more prominent expressions of either Groupthink or Polythink symptoms in the teams and at the same time push them away from the range of Con-Div at the center of the continuum. Based on the characteristics of medical teams working together in stressful emergency life-threatening conditions [8,9,10,13] we assumed in our third hypothesis (H3) that exposure to rudeness will have a more prominent effect on enhancing Groupthink symptoms. Regarding the second and third hypotheses (H2 & H3), it appears that exposure to rudeness did not affect the group decision-making dynamics of the NICU teams. There were no significant differences between the control and rudeness groups. Therefore, it seems that exposure to rudeness does not lead to more prominent expressions of either Groupthink or Polythink symptoms in the teams. The scores of the different teams examined in this study did not show a consistent tendency towards Groupthink or Polythink, as expressed by the General Score, which was close to the center of the continuum, i.e., Con-Div, which might even be considered an optimal situation in certain cases. In conclusion, because no significant differences were found, it seems that exposure to rudeness does not affect the group dynamics of NICU teams’ decision-making process. 

Regarding the methodology we employed, our conclusion is that the new quantitative assessment tool we developed, consisting of 14 items, was a useful, feasible and reasonably reliable research tool to be further studied in medicine and other disciplines engaged in decision-making. Although the measure we were using seems to be promising, we must address its possible limitations. Previous research in social psychology and organizational behaviour raised concerns regarding Groupthink as a construct measure when it was empirically tested [14,15,16,17]. The main problem is the definition of symptoms, which strongly implies that symptoms point to a single, unitary, underlying condition. However, there is no empirical evidence that Groupthink is actually a unitary, underlying thing. Groups can only be evaluated on the collection of practices they employ, and these are not necessarily correlated with each other: How well do teams combine diverse composition with processes that tap diverse thinking? The label “Groupthink” reifies a unitary condition that may not in fact exist. A different way to think about groups might be as a combination of composition (diverse or not) and processes (norms and techniques that allow the sharing and tapping of diversity). Groups may also be transitioning through phases, from having a period of early divergence (avoiding premature closure) to achieving convergence later on. In this study, we tried to address these limitations by using a continuum between Groupthink and Polythink, and by using a quantitative-based measure that allowed for different degrees in each of the symptoms. Thus, we believe that the current measure we suggest is an important step in the direction of trying to measure aspects of group process. However, this measure might call for improvements with further studies. More constructs might be added including: relational conflict, which is generally bad vs. task conflict which is usually good [18,19,20]; the role of trust and psychological safety in encouraging people to speak up [21,22]; and the role of participative leadership in creating an open environment for sharing information (as opposed to autocratic or directive leadership) [23]. In addition, the separation of Groupthink and Polythink extreme dimensions implied a linear graph as shown in Figure 1. However, it is possible that future developments of our suggested model could imply more than a two-dimensional space model, because optimal performance could have more than two dimensions. Best teams could show for example cohesion (a symptom of Groupthink) because they trust and respect each other, but use this cohesion to have a ‘good fight’ if diverse views are present (due to diverse composition of experience and training). Thus, further studies could lead to a more than two-dimensional measure for group dynamics performance.

We believe that the concepts of Groupthink, Polythink, and Convergence-Divergence are important factors for diagnosing optimal and suboptimal decision-making in groups in general, and they are highly relevant for groups in the medical domain. Our attempt to develop a new tool for this applied context is thus important despite all the above-mentioned limitations.

The study itself also has several limitations. Even though the study included 24 NICU teams, recorded participating in a simulation of approximately 40 min each, the size of the sample might still have been too small to give enough insights regarding the suggested tool’s ability to expose subtle differences in medical teams’ group dynamics. Perhaps testing the tool on a bigger sample size will uncover clearer tendencies towards Groupthink or Polythink. Additionally, the number of judges in this study might have been insufficient, even though in the original research there were also three judges who similarly used assessment tool with a 5-point rating scale to evaluate the videotapes [4].

Another possibility is that watching videotapes and evaluating the teams accordingly in retrospect, and not in real time as the simulations are taking place, made it difficult for the judges to examine each team’s decision-making process and group dynamics. Assessing group dynamics in decision-making is different from the original study, where the judges were asked to evaluate the teams’ performance, which was probably easier to do by only watching their recordings [4]. A possible solution is to consider adjusting the design in order to allow team members, participating in similar simulations, to evaluate their own team’s group dynamics using the same assessment tool. These could then be compared to evaluations by external judges, the latter preferably rated in real time.

We suspect that because Groupthink is a well-known and established risk factor for medical teams [8,9,10,13], while Polythink is a relatively new concept [11,12], agreement among raters for Polythink was lower than for Groupthink. A trial of a shorter 10 items assessment tool based on CFA loading estimates did not result in a better model fit, and thus we suggest that currently the 14 items assessment tool should be the one to be further studied.

Another interesting question relates to the possibility that any external input from someone could have been interpreted as rudeness instead of disruption and vice versa. Disruption of thought is not wrong if it is a protest at pending error (defined as ‘speaking up’ which we try to encourage in medical teams). The question is how can we separate in these cases unity of thought from coercion or conformity? It is well known that such groupthink if blind to a particular factor can be the road to disaster in clinical settings. Our data cannot provide a definite answer to this dilemma, although we have tried to overcome it in our instructions to the judges regarding their assessments using the tool. The fact that our teams were close to the Con-Div center of the group decision-making dynamics continuum regardless of their exposure to rudeness also precludes such possibility. However, since the judges were blinded to the primary rudeness incident itself this should still be regarded as a limitation. 

This study is unique because it made the first attempt at using quantitative research methods to test the theories in the field of decision-making dealing with the different types of group dynamics, by adapting the symptoms described in the literature for assessment in a structured assessment tool. This is in contrast to previous studies that used qualitative methods to test group dynamics’ theories. Indeed, it could be argued that the opinions of the raters are still subjective, which limits the objectivity of the result. However, these quantitative assessments following accurate definitions with good training of the judges in advance, allow for a quantitative statistical analysis as opposed to using only qualitative impressions.

This study also examined the group decision-making dynamics of medical teams. This is the first study to identify intra-group dynamics beyond Groupthink, and apply it to Polythink and Con-Div, using a quantitative method. In light of the encouraging reliability (internal consistency and agreement among raters) and the supportive CFA, it seems that there is a good basis to continue developing and using the quantitative tool presented in this study in order to assess group dynamics in additional studies, whether in the medical field or other disciplines engaged in decision-making.

## Figures and Tables

**Table 1:** Raters’ assessment tool for group dynamics in medical teams. Please indicate the level of agreement or disagreement with each of the following 14 statements, by circling the number best describing your opinion\feeling regarding the team you just watched. The scale is from 1 (Strongly disagree) to 5 (Strongly agree).

	Strongly Disagree	Disagree	Neither Agree nor Disagree	Agree	Strongly Agree
1. Confusion and lack of communication (intentional lack of communication, mixed messages from different members, inadvertent failures to communicate effectively)	1	2	3	4	5
2. Collective efforts to rationalize in order to discount warnings and negative feedback which might lead the members to reconsider their assumptions (post hoc selection of facts and arguments to rationalize a course of action already chosen)	1	2	3	4	5
3. No room for reappraisal of previously rejected options (not because of overwhelming consensus, but because of unwillingness to reopen and discuss previously reconciled arguments which may be time consuming)	1	2	3	4	5
4. Greater likelihood of framing effects (some members may advance biased or selective information to promote their views at the expense of others- for example, what they prefer will be framed in a positive light and what they oppose in a negative light)	1	2	3	4	5
5. Self-censorship by group members such that doubts are not shared publicly (members silence themselves and even minimize the importance of their misgivings and counterarguments so as not to harm the group consensus)	1	2	3	4	5
6. Adoption of positions with the lowest common denominator. The group might settle on a compromise option that tries to partially satisfy all or most members. Each member needs to make some concessions or compromises (Stronger members might marginalize or expel those with competing views, thereby imposing their own on the group)	1	2	3	4	5
7. Greater likelihood of conflict within the group (because of differing and even opposing opinions and solutions)	1	2	3	4	5
8. A shared illusion of unanimity (partly from the false assumption that whoever remains silent supports the majority’s judgments)	1	2	3	4	5
9. Limited review of options (the debate may become unmanageable, therefore often some options will be quickly excluded from consideration so as to remain with a smaller choice set for more thorough consideration)	1	2	3	4	5
10. Overinflated view of the competence of the group (illusion of invulnerability- provides reassurance about obvious threats, leads to over optimism and willingness to take extraordinary risks)- overconfidence	1	2	3	4	5
11. Direct pressure on any member who expresses doubt in the group’s assumptions or questions the validity of the arguments supporting the favored action plan, making clear that this is contrary to what is expected of a loyal team member (demands for conformity)	1	2	3	4	5
12. Self-appointed “mindguards”- members who keep from the group expert or opposing information that might call into question its course of action	1	2	3	4	5
13. Decision paralysis (failure to act, stem or prevent potential crises, or the adoption of suboptimal, satisficing solutions that are often short-sighted and inhibit long term planning) *	1	2	3	4	5
14. A belief in the moral rightness or ethical superiority of the group members compared with anyone who might challenge their course of action **	1	2	3	4	5

* One symptom of Polythink—higher chance of leakage of information—was omitted from the assessment tool. In Polythink, there is a greater chance that information will leak out because of group members’ attempts to undermine positions they oppose [11,12]. As the simulations examined in the study included only the participants of each team and there was no interaction with other outside sources, this symptom was irrelevant. We attempted to adapt this symptom to the circumstances of the team simulations the judges reviewed. The item was thus formulated: “Members constantly comment and express opinions that aim to undermine positions and actions they oppose”. Due to the judges’ difficulty in evaluating this item, and its relative irrelevance to the medical simulations’ scenarios, it was removed at an early stage of the analysis (item number 14 which does not appear here). The first confirmatory factor analysis (CFA) with the 15 items confirmed that it was not related to Polythink. ** One symptom of Groupthink—disparaging stereotypes of any opposing sources outside the group—was also omitted for similar reasons. Therefore, the original assessment tool consisted of 8 symptoms for Polythink (number 14 later removed as outlined above) and 7 symptoms for Groupthink, presented in the same mixed and random order to the different judges.

**Table 2:** Inter-rater agreement and confirmatory factor analysis testing 2-factor model (Polythink and Groupthink) for the items of the assessment tool examining group dynamics in medical teams.

	Item	ICC(1)	[95% CI]	Confirmatory Factor Analysis		
**Polythink**		**0.66**	**0.46, 0.82**	**Factor Loading**		**R^2^**
Confusion and lack of communication	1	0.50	0.25, 0.71	1.000	<0.001	0.408
No room for reappraisal of previously rejected options	3	0.53	0.29, 0.73	1.289	<0.001	0.678
Greater likelihood of framing effects	4	0.68	0.48, 0.83	0.491	0.005	0.098
Adoption of positions with the lowest common denominator	6	0.76	0.58, 0.88	1.116	<0.001	0.508
Greater likelihood of conflict within the group	7	−0.01	−0.2, 0.27	0.436	0.010	0.078
Limited review of options	9	0.81	0.66, 0.90	1.421	<0.001	0.824
Decision paralysis	13	−0.33	−0.41, −0.17	0.940	<0.001	0.360
**Groupthink**		**0.82**	**0.69, 0.91**	**Factor Loading**		**R^2^**
Collective efforts to rationalize	2	0.20	−0.04, 0.48	1.000	<0.001	0.389
Self-censorship by group members	5	0.67	0.46, 0.83	0.948	<0.001	0.349
A shared illusion of unanimity	8	0.25	0.0, 0.52	0.478	0.005	0.089
Overinflated view of the competence of the group—overconfidence	10	0.79	0.63, 0.89	1.021	<0.001	0.405
Direct pressure on any member who expresses doubt—demands for conformity	11	0.84	0.71, 0.92	1.075	<0.001	0.449
Self-appointed “mindguards”	12	0.84	0.71, 0.92	0.741	<0.001	0.213
A belief in the moral rightness or ethical superiority of the group members	15	0.51	0.27, 0.73	0.437	0.006	0.074

ICC(1)—Intraclass Correlation Coefficient 1.

**Table 3:** The effects of exposure to rudeness on NICU teams’ group dynamics in decision-making.

Variable	Control Group (*n* = 11)		Rudeness Group (*n* = 13)		*t*-Test	*p*-Value
	Mean	SD	Mean	SD		
Polythink	2.88	0.48	2.83	0.49	−0.25	0.80
Groupthink	2.75	0.34	2.73	0.41	−0.16	0.88
General Score	0.13	0.45	0.10	0.42	−0.15	0.88

## Data Availability

The data presented in this study are available on request from the corresponding author.
